# A Triple Regimen as an Alternative Treatment for Lymphatic Filariasis in a Non-endemic Area in Indonesia: A Case Report

**DOI:** 10.7759/cureus.92576

**Published:** 2025-09-17

**Authors:** Alindina Anjani, Rizka Humardewayanti Asdie

**Affiliations:** 1 Division of Tropical Infection, Department of Internal Medicine, Dr. Sardjito General Hospital/Faculty of Medicine, Public Health and Nursing, Universitas Gadjah Mada, Yogyakarta, IDN; 2 Internal Medicine, Dr. Sardjito General Hospital, Yogyakarta, IDN

**Keywords:** case report, drug therapy, lymphatic filariasis, treatment failure, triple drug regimen

## Abstract

Lymphatic filariasis is a neglected tropical disease. Although cases in Indonesia are not very common, the condition still poses a burden because of its chronic course and the disability it causes in daily life. Research on treatment is limited, especially for patients who do not respond to standard regimens in non-endemic areas. We report the case of a 19-year-old male with a four-year history of bilateral non-pitting edema of the lower limbs. He had no prior medical history and was diagnosed with filariasis of undetermined species because of limited laboratory capacity for species identification. Because diethylcarbamazine (DEC) was not available at the time, the patient was first treated with albendazole 400 mg twice daily for 14 days and a single dose of ivermectin 36 mg. Thirty days later, he returned with persistent edema, and blood smear examination still showed microfilaria. A second regimen consisting of single-dose albendazole 400 mg and DEC 600 mg daily for 12 days led to partial improvement in edema, although microfilariae were still detected. A third regimen was then administered: albendazole 400 mg twice daily for 21 days, DEC 600 mg daily for 12 days, and a single dose of ivermectin 48 mg, along with supportive lymphedema care. Over 90 days, his edema gradually improved, and no adverse effects were reported. A triple regimen of albendazole, DEC, and ivermectin was effective in a patient who failed the initial albendazole-ivermectin regimen. This approach may be considered in similar cases, but further studies are needed to explore species-specific resistance and guide treatment.

## Introduction

Filariasis is a neglected tropical disease that continues to be a public health problem in Indonesia, even though it occurs mainly in specific endemic regions [1]. Globally, Southeast Asia accounts for about 50% of the world’s 120 million cases of filariasis [2]. In Indonesia, 10,681 cases of lymphatic filariasis were reported in 2019, 70% of which were due to *Brugia malayi* [3]. By 2022, 21 districts or cities still reported ongoing cases and were undergoing mass treatment, 178 districts or cities were in the post-treatment surveillance stage, and 37 districts or cities had been declared filariasis-free by the Indonesian Minister of Health [4]. A district or city is classified as filariasis-free if it fulfills the following criteria: (1) successfully implementing the Mass Preventive Drug Administration (MDA) program for five consecutive years, (2) achieving a microfilariae prevalence of less than 1% in sentinel and spot-check villages after the last effective MDA round, and (3) passing the transmission assessment survey on three occasions [5].

Lymphatic filariasis in Indonesia is caused by three species of nematodes: *Wuchereria bancrofti*, *Brugia timori*, and *Brugia malayi* [6]. Currently, 23 mosquito species from five genera in Indonesia are capable of transmitting filarial parasites [5]. Infection can present in asymptomatic, acute, or chronic forms, with affected individuals sometimes developing lymphadenopathy that can be disabling, although the disease rarely causes death [7]. Lymphatic filariasis often leads to significant morbidity due to lymphatic damage, causing swelling and disability that affect patients’ quality of life. While effective treatment typically halts disease progression, treatment failure remains a critical concern impacting patient outcomes and elimination programs.

There is no literature from Indonesia recommending a treatment regimen for lymphatic filariasis cases in non-endemic areas that fail standard treatment, which consists of either diethylcarbamazine (DEC) 6 mg/kg/day for 12 days, ivermectin 200 µg/kg as a single dose, or albendazole 400-800 mg daily for two to three weeks [8]. We report here a case of a patient who failed initial treatment but responded to an alternative regimen. We received ethical clearance to report this case from the Ethics Committee of the Faculty of Medicine, Health Science, and Nursing, Gadjah Mada University (KE/FK/0354/EC/2023). The patient gave written informed consent to report his case.

## Case presentation

The patient was a 19-year-old male college student from a non-endemic area for filariasis who presented to the clinic with a four-year history of bilateral lower-extremity edema, more severe in the left leg. The edema began in the feet and gradually progressed proximally. He reported no visual or urinary symptoms and had never taken anti-filarial medication. His past medical and family history were unremarkable.

On physical examination, he had bilateral non-pitting edema of the lower extremities, graded as grade 3 in the right leg and grade 4 in the left leg, extending to the inguinal regions bilaterally. Both legs demonstrated skin folds, mossy lesions, hyperpigmented plaques, hypopigmented patches, and scaling.

**Figure 1:**
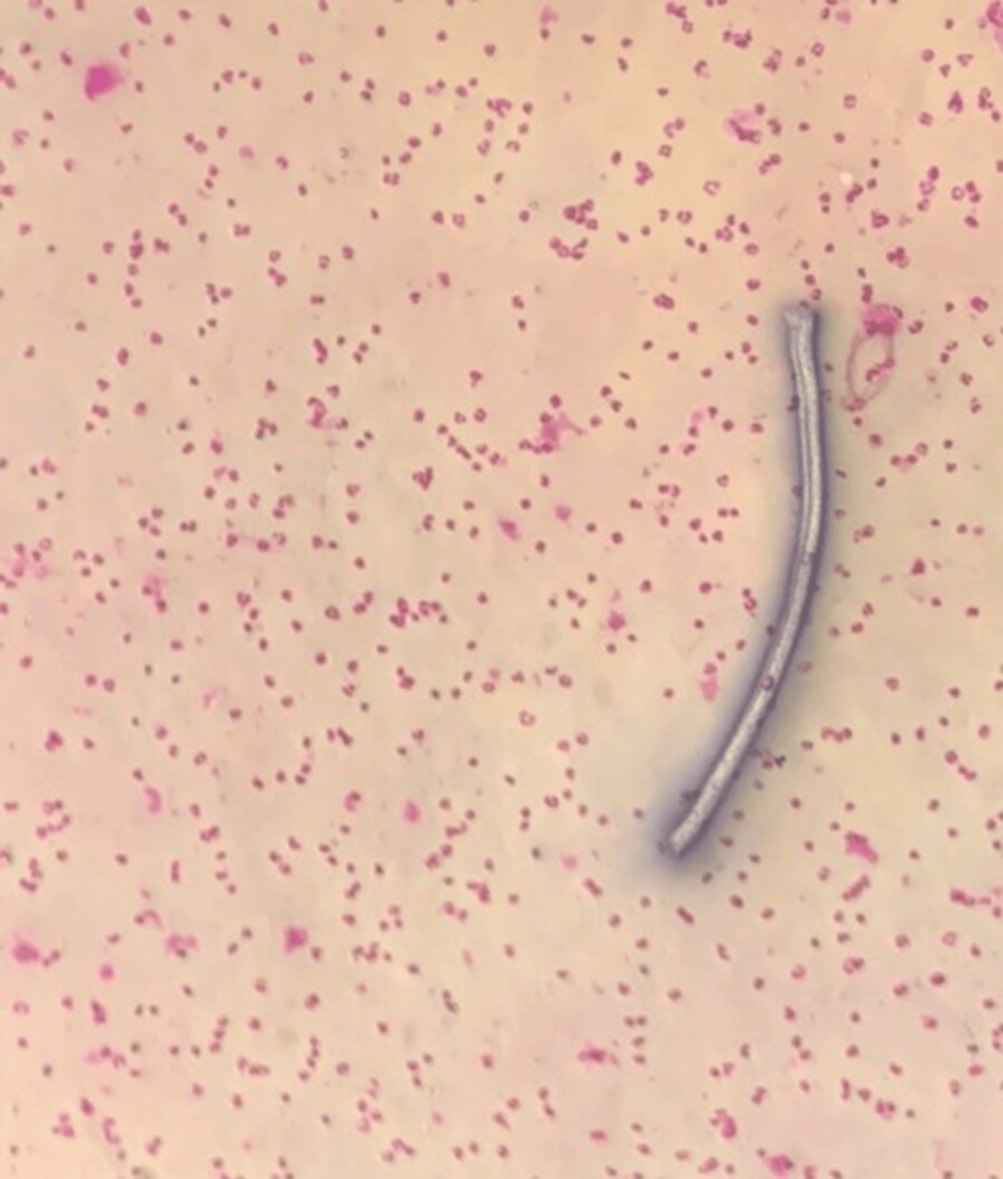
Photomicrograph of a microfilaria in the peripheral blood smear (10x magnification).

**Figure 2:**
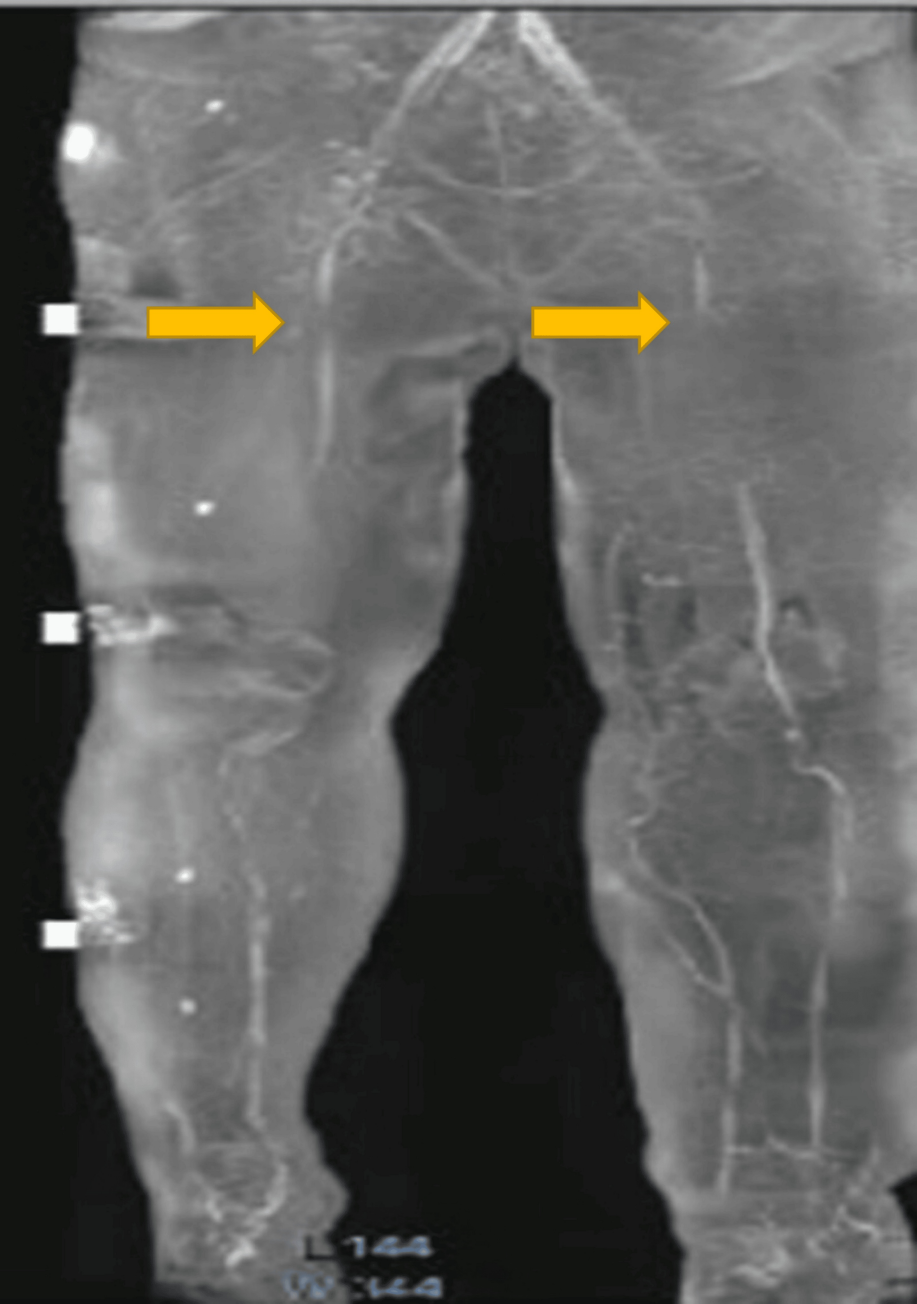
Magnetic resonance lymphangiogram (lymph-sequence) of the patient’s bilateral lower extremities showing obstruction below the inguinal lymph nodes, indicated by the arrows.

There was no clinical history or examination findings suggestive of hypothyroidism, heart failure, kidney disease, or liver disease. A complete blood count showed elevated eosinophils and renal function tests were within normal limits (Table 1). Microscopic examination of the peripheral blood smear revealed an average of 13 microfilariae per field at 10× magnification (Figure 1). The species could not be identified due to limited laboratory expertise. Ultrasound of the lower extremities showed no thrombus or filarial dance sign, while contrast-enhanced magnetic resonance lymphangiography demonstrated bilateral lower-extremity lymphedema, more severe on the left (Figure 2).

**Table 1:** Baseline laboratory result MCV: Mean Corpuscular Volume, MCH: Mean Corpuscular Haemoglobin, BUN: Blood Urea Nitrogen

Parameters	Result	Unit of Measurement	Normal Reference Value
Erythrocyte	4.97	10^6/µL	4.60 - 6.00
Haemoglobin	13.6	g/dL	14.0 - 18.0
Haematocrit	40.4	%	40.0 - 54.0
MCV	81.2	fL	80.0 - 94.0
MCH	27.3	pg	26.0 - 32.0
Leucocyte	7.8	10^3/µL	4.50 - 11.50
Neutrophile	58.9	%	50.0 - 70.0
Lymphocyte	28.2	%	18.0 - 42.0
Monocyte	4.3	%	2.0 - 11.0
Eosinophile	8.3	%	1.0 - 3.0
Basophile	0.3	%	0.0 - 2.0
BUN	6.00	mg/dL	6.00 - 20.00
Creatinine	0.88	mg/dL	0.70 - 1.20

He was diagnosed with lymphatic filariasis and was initially treated with albendazole 400 mg twice daily for 14 days, followed by a single 36-mg dose of ivermectin, given in place of DEC due to limited drug availability. At follow-up, 30 days after the initial regimen, a peripheral blood smear still showed one microfilaria. He was then treated with albendazole 400 mg (single dose) combined with DEC 600 mg daily for 12 days. Thirty days after the second regimen, the blood smear continued to show one intact microfilaria and three microfilarial fragments. A third regimen was administered: albendazole 400 mg twice daily for 21 days, DEC 600 mg daily for 12 days, and a single 48-mg dose of ivermectin. Follow-up at 30 days after the third regimen showed a negative peripheral blood smear.

**Figure 3:**
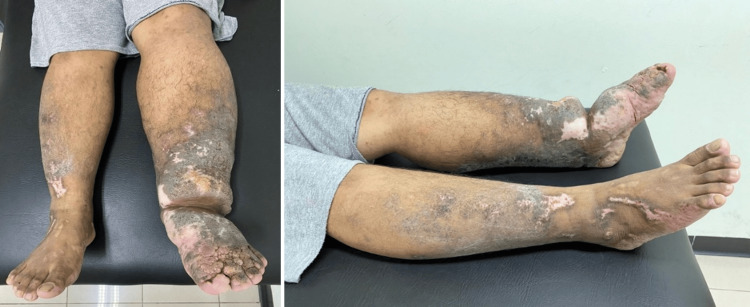
Photograph of patient’s lower extremities 90 days after initial treatment.

Throughout treatment, the patient was also educated to use compression stockings and elastic bandages, perform lymphedema exercises and lymphatic massage, elevate the affected limbs, and maintain skin hygiene to prevent secondary infection. Non-pitting edema began to improve after the second regimen, with no evidence of secondary infection. Over 90 days, his symptoms improved gradually, with edema reduced to grade 2 in the right leg and grade 3 in the left leg (Figure 3). Residual swelling persisted, and the patient continued adherence to physiotherapy.

## Discussion

Standard anti-filarial therapy for cases of lymphatic filariasis in Indonesia is one of three drugs: DEC, ivermectin, or albendazole [8]. DEC works by inhibiting the cyclooxygenase pathway, causing microfilariae immobility and apoptosis due to microfilarial DNA damage and a macrofilaricidal effect. A single dose of DEC can kill macrofilariae that are sensitive to this drug, but in cases of DEC resistance, repeated doses have no effect on macrofilaria [9]. Ivermectin affects the microfilariae for glutamate-gated chloride channels, causing parasite paralysis and death. Ivermectin has no proven effect on macrofilariae. Albendazole binds to filarial beta-tubulin, inhibiting the formation of microtubule matrices, stopping cell division, inhibiting glucose uptake and suppressing reproduction, resulting in immobilization and death of macrofilariae but does not have a direct effect on microfilariae. However, albendazole 400 mg to treat macrofilariae in combination with DEC or ivermectin to treat microfilariae results in greater efficacy [9,10].

In non-endemic areas of Indonesia there is limited access to DEC and ivermectin. This results in albendazole being the drug of first choice to treat filariasis; however, substantial evidence supporting the limited efficacy of albendazole alone in eradicating microfilaremia led to the administration of a combination therapy incorporating ivermectin in this case [11]. The recommended dose of ivermectin for filariasis therapy in Indonesia is 200 mcg/kg body weight, while previous research states that a dose of 400 mcg/kg body weight shows better parasite clearance results [12]. Ivermectin is approved in Indonesia to treat only strongyloidiasis or onchocerciasis [13]. Limited access to ivermectin resulted in a dose of 300 mcg/kg body weight being given to this patient.

In our patient’s case, microfilariae were still detected in this patient’s peripheral blood after being given a combination of albendazole and ivermectin, raising the possibility of drug resistance. Filarial resistance to DEC has been reported from India and ivermectin resistance has been reported from Ghana [10,14]. We are not able to do resistance testing at our hospital but it is presumed in our case report. Therefore, we treated this patient with a triple drug regimen (ivermectin, DEC and albendazole (IDA)).

Several studies have compared the effectiveness of IDA with the combination of ivermectin and albendazole (IA) and the combination of DEC and albendazole (DA). A study from Papua New Guinea showed IDA was 4.5 times more effective than DA to clear microfilariae [15]. A study from Cote d'Ivoire found that a single dose of IDA was more effective than two doses of IA at 24 months [16]. A study from Indonesia reported IDA was more effective at clearing microfilariae than DA [17]. In our reported case, IDA successfully cleared microfilariae from peripheral blood smears and no side effects of this regimen were noted.

In addition to drug treatment, it is important to use other modalities to manage the effects of lymphedema [18]. Our reported patient was shown how to use compression stockings and elastic bandage, how to do physiotherapy such as self-massage, lymphedema exercise, and routine elevation of the affected limbs, and to maintain good skin hygiene to prevent secondary bacterial infections. At the last visit, the edema was objectively reduced. There was a decrease in the degree of edema from grade 3 to grade 2 for the right leg, and grade 4 to grade 3 for the left leg, and the patient was able to wear closed footwear.

## Conclusions

The observed clinical improvement following the second treatment regimen suggests a potential alternative approach for cases unresponsive to standard therapy. However, as these findings are based on a single case, further evidence is needed to support their generalizability. Identifying the infecting species may help clarify whether treatment failure is associated with species-specific drug resistance.
